# The Curative Influence of Exploratory Incisions

**Published:** 1898-03-12

**Authors:** 


### THE CURATIVE INFLUENCE OF EXPLORATORY INCISIONS.

To prescribe "an operation" without any definite idea as to what it is that the operation is to do or to remove, seems an uncouth and somewhat empirical proceeding. Yet there seem to be cases in which surgical intervention does good although the surgeon may find it quite impossible to say in what way the good arises. This has long been recognised in regard to laparotomy, cases occurring in which the operation appears to effect either the cure of a disease or at least its temporary amelioration, even though, so far as appearance goes, little may have been done beyond opening the abdomen and sewing it up again. In a recent paper read before the Medical Society, Mr. Treves discussed the subject of Abdominal Section as a Medical Measure. Prominent among the conditions for the relief of which a simple laparotomy has been useful is tuberculous peritonitis. "The results of the treatment of this disease by simple incision have been little short of miraculous." The large series of cases compiled by Aldibert, dealing with 308 examples of this form of peritonitis treated by operation, shows a percentage of cures estimated at 69·8 per cent., of which number 33·4 per cent. may be regarded as complete recoveries, and an examination of this record shows that the most favourable results have, on the whole, been those attending the mere opening of the abdomen. Sometimes the diagnosis has been made of an ovarian cyst or of some other such growth. The surgeon has proceeded to open the abdomen to remove the hypothetical tumour, has discovered simply tuberculous peritonitis, has hastily closed the incision, and has gone away under the impression that his operation has been useless, if not injurious. In due course, however, he has heard to his amazement that the patient has made an excellent recovery. In some cases more has been done; the peritoneal cavity has been washed out, or drained, or some such medicament as iodoform has been introduced, and the surgeon has thus been able to take credit to himself for the part that he has played; but curiously enough, except in suppurative cases, these elaborate measures have not been attended with such good results as have followed the mere incision made by mistake.

Indeed, the incision made by mistake, says Mr. Treves, can claim some of the most brilliant achievements of surgery in connection with tuberculous peritonitis. How such operations act it is impossible to say.

Other cases are met with in which a mere incision into the peritoneal cavity has led to the rapid shrinking of certain malignant growths, and to the temporary improvement of the patient. In most of these a tumour of doubtful nature has been discovered, the abdomen has been opened, the growth has proved to be a sarcoma, and nothing has been done. After the operation the mass has dwindled in size, and in some cases seems to have vanished—certainly for a time.

Again, in that large class of cases somewhat hopelessly styled "nervous," relief sometimes unexpectedly follows abdominal section, with or without some further operative procedure. Some of these are cases of nervous mimicry, the mimicry going so far as to lead to recovery after operation! In others the clinical phenomena are simply bizarre and fantastic; nevertheless, restoration to health has sometimes followed operation, as, for example, where it has been done in cases in which hysterical vomiting has led to such exhaustion as to lead to the suspicion of organic disease.

After passing in review cases of many kinds in which cure has resulted after exploratory incision, Mr. Treves gives a warning which seems to us of some importance. He says: That this simple procedure has been of enormous value no one will doubt; that it has been the means of saving many a life has been amply demonstrated; that it has enabled a correct diagnosis to be made, and a logical treatment to be carried out in hundreds of obscure cases needs not to be insisted on; but there must arise in the minds of many the question whether the exploratory incision, infinite as its value may be, is an entirely unmixed blessing. I notice that there are indications of a tendency to allow this ready measure to replace the admirable labour of clinical observation. The incision is so simple; the collecting and arranging and judging of clinical evidence is so difficult and tedious. With a scalpel in the hand, the patient searching examination of the abdomen, as practised in old days, is no longer needed; and it is a question whether the education of those who wish to become acute clinical observers has not suffered a little thereby.

